# Participation in Physical and Sportive Activities among Adult Turkish People with Hemophilia: A Single-Center Experience

**DOI:** 10.4274/tjh.2017.0292

**Published:** 2018-03-06

**Authors:** Arni Lehmeier, Muhlis Cem Ar, Sevil Sadri, Mehmet Yürüyen, Zafer Başlar

**Affiliations:** 1 İstanbul University Cerrahpaşa Faculty of Medicine, Department of Internal Medicine, Division of Hematology, İstanbul, Turkey; 2 İstanbul University Cerrahpaşa Faculty of Medicine, Department of Internal Medicine, Division of Geriatrics, İstanbul, Turkey

**Keywords:** Hemophilia, Physical activity, Sports, Turkey

## To the Editor,

Because of the increased bleeding risk, people with hemophilia (PwH) were advised to avoid physical activity (PA) until the 1970s [1]. However, with the advent of modern treatment modalities and regarding the numerous benefits that PA offers, currently PwH are encouraged to participate in PA and sports as much as possible [2]. Nevertheless, how physically active are adult PwH? This question has readily been studied in high-income countries with unrestricted access to coagulation factors [3], but facts are lacking about the awareness level on PA and its prevalence among adult hemophiliacs in developing countries, such as Turkey, where the market entrance of coagulation factors and the practice of prophylaxis have been relatively recent.

In order to assess this question, 70 Turkish PwH with hemophilia A (84.3%) and B (15.7%) aged 19-61 years (mean: 38.0±11.8) were asked to complete a questionnaire that included questions on the sociodemographic characteristics and bleeding patterns of the patients, their attitudes towards exercise and sports, and their levels of involvement in PA.

The study strikingly showed that Turkish PwH had a low level of awareness about PA. Less than one-fifth of the patients reported being sufficiently involved in PA. The level of involvement was highest (35%) in young adults (18-29 years) and lowest (0%) in patients aged 50-69 years (p<0.05). Conversely, in a German study [4], more than half of the adult hemophiliacs were very interested in exercise and sports. Although sportive activity is not equal to PA, German PwH seem to be more involved in an active lifestyle than Turkish patients.

However, the present results might be associated with the severity of hemophiliac arthropathy, which is significantly more prevalent in older age groups (p<0.05).

Despite the low awareness of PA, more than 40% of the patients met the current World Health Organization recommendations for PA [5], with young adults (65%) again being significantly more involved in physical activities than older PwH (23%) (p<0.05).

Our results indicate that most of the patients avoid sportive activities (60%). Those who are physically active reported preferring moderate-intensity PA like walking or climbing stairs, instead of vigorous-intensity activities. As expected, the level of sportive activity significantly declined with increasing age (p<0.05).

What are the reasons underlying the low level of PA in adult PwH in Turkey? Pain, fear of being injured, and lack of motivation were the most frequently reported reasons for avoiding PA. This is not surprising, given the late implementation of prophylaxis in Turkey (in 2005) and the resultant high prevalence of hemophilic arthropathy among elderly Turkish PwH causing pain and immobility.

A multidisciplinary approach for implementation of suitable/safe physical exercises associated with less or no pain would help patients overcome the fear of being injured and thus increase their involvement in PA. The risk of injury can be minimized by following the recommendations for safe PA for hemophiliacs [6], which are often ignored, as shown by the patients (Table 1).

In conclusion, a reasonable treatment program for hemophilia should include much more than just factor replacement. PwH should be educated on the positive impact of PA on their physical, social, and psychological well-being. Furthermore, they should be well instructed about the recommendations for safe PA and what happens if they ignore the safety issues. PA should be considered as an integrated part of modern hemophilia treatment, which requires the collaboration of experts from various scientific fields.

## Figures and Tables

**Table 1:**
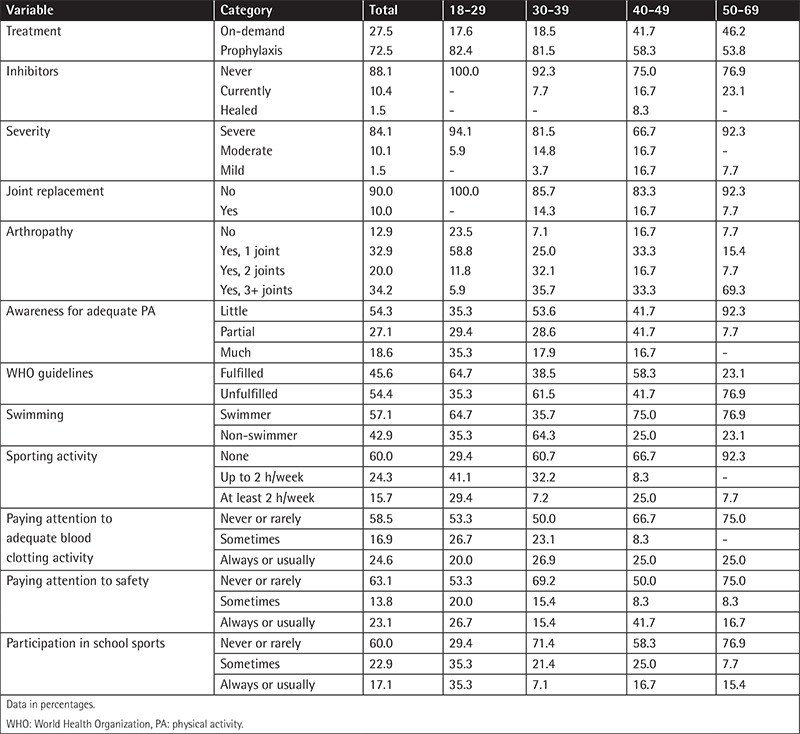
Results of the questionnaire according to age groups.
